# JCHLA/JABSCS Special Issue on the COVID-19 pandemic and the health information profession

**DOI:** 10.29173/jchla29679

**Published:** 2023-04-01

JCHLA/JABSC invites submissions for the Special Issue to be published in April 2024. This Special Issue strives to document the challenges and innovations of health information professionals during the COVID-19 pandemic. This Issue will highlight the tireless work of information professionals during this critical time and also provide an important evidence base for potential future pandemics.

Please feel free to contact the Editor-in-Chief (editor@chla-absc.ca) with proposals or to confirm that your submission meets the scope of JCHLA/JABSC and the Special Issue.

Potential topics include, but are not limited to:

- Shift to virtual learning spaces
- Literature search services/challenges for COVID-19 evidence
- Information management systems used for COVID-19 information dissemination
- Experiences of redeployment within health organizations
- Consumer information seeking for COVID-19 information
- Closures of physical library spaces

Peer-reviewed submissions (i.e. Research Articles, Program Descriptions and Literature Reviews) must be submitted by the end of day September 30, 2023. Non-peer-reviewed submissions (i.e. Columns and Comment and Opinion) must be submitted by January 7, 2024. Please see the Author Guidelines for details for each submission type.

Please contact editor@chla-absc.ca with any questions.

## Numéro spécial du JABSC / JCHLA sur la pandémie de COVID-19 et la profession d'information sur la santé

JABSC / JCHLA sollicite des soumissions pour le numéro spécial qui sera publié en avril 2024. Ce numéro spécial s'efforce de documenter les défis et les innovations des professionnel(-le)s de l'information sur la santé pendant la pandémie de COVID-19. Ce numéro mettra en lumière le travail inlassable des professionnel(-le)s de l'information pendant cette période critique et fournira également une base de données importante pour les futures pandémies potentielles.

N'hésitez pas à contacter la rédactrice en chef (editor@chla-absc.ca) pour lui faire part de vos propositions ou pour confirmer que votre soumission correspond au champ d'application de JABSC / JCHLA et du numéro spécial.

Les sujets potentiels incluent, mais ne sont pas limités à :

- Le passage à des espaces d'apprentissage virtuels
- Services de recherche documentaire / défis pour les données probantes relatives à la COVID-19
- Systèmes de gestion de l'information utilisés pour la diffusion des informations relatives à la COVID-19
- Expériences de redéploiement au sein des organismes de santé
- Recherche d'informations sur la COVID-19 par les consommateurs
- Fermeture de bibliothèques physiques

Les soumissions évaluées par les pairs (c'est-à-dire les articles de recherche, les descriptions de programmes et les analyses documentaires) doivent être soumises avant la fin de la journée du 30 septembre 2023. Les articles non évalués par des pairs (c'est-à-dire les colonnes et les commentaires et opinions) doivent être soumis au plus tard le 7 janvier 2024. Veuillez consulter les lignes directrices à l'intention des auteur(-e)s pour plus de détails sur chaque type de soumission.

Pour toute question, veuillez contacter editor@chla-absc.ca.

